# Dysfunctional high-density lipoproteins have distinct composition, diminished anti-inflammatory potential and discriminate acute coronary syndrome from stable coronary artery disease patients

**DOI:** 10.1038/s41598-017-07821-5

**Published:** 2017-08-04

**Authors:** Mihaela G. Carnuta, Camelia S. Stancu, Laura Toma, Gabriela M. Sanda, Loredan S. Niculescu, Mariana Deleanu, Andreea C. Popescu, Mihaela R. Popescu, Adelina Vlad, Doina R. Dimulescu, Maya Simionescu, Anca V. Sima

**Affiliations:** 1 Institute of Cellular Biology and Pathology “Nicolae Simionescu” of the Romanian Academy, Bucharest, Romania; 2 University of Agronomical Sciences and Veterinary Medicine Bucharest, Faculty of Biotechnology, Bucharest, Romania; 3 Cardiology Clinic, Elias University Hospital, Bucharest, Romania; 4 Physiology Department, “Carol Davila” University of Medicine and Pharmacy, Bucharest, Romania

## Abstract

There is a stringent need to find means for risk stratification of coronary artery diseases (CAD) patients. We aimed at identifying alterations of plasma high-density lipoproteins (HDL) components and their validation as dysfunctional HDL that could discriminate between acute coronary syndrome (ACS) and stable angina (SA) patients. HDL_2_ and HDL_3_ were isolated from CAD patients’ plasma and healthy subjects. ApolipoproteinAI (apoAI), apoAII, apoCIII, malondialdehyde (MDA), myeloperoxidase (MPO), ceruloplasmin and paraoxonase1 (PON1) were assessed. The anti-inflammatory potential of HDL subfractions was tested by evaluating the secreted inflammatory molecules of tumor necrosis factor α-activated endothelial cells (EC) upon co-incubation with HDL_2_ or HDL_3._ We found in ACS versus SA patients: 40% increased MPO, MDA, apoCIII in HDL_2_ and HDL_3_, 35% augmented apoAII in HDL_2_, and in HDL_3_ increased ceruloplasmin, decreased apoAII (40%) and PON1 protein and activity (15% and 25%). Co-incubation of activated EC with HDL_2_ or HDL_3_ from CAD patients induced significantly increased levels of secreted inflammatory molecules, 15–20% more for ACS versus SA. In conclusion, the assessed panel of markers correlates with the reduced anti-inflammatory potential of HDL subfractions isolated from ACS and SA patients (mostly for HDL_3_ from ACS) and can discriminate between these two groups of CAD patients.

## Introduction

Coronary artery disease (CAD) is one of the primary causes of death worldwide and includes stable angina (SA) and acute coronary syndrome (ACS)^1^. The main cause of CAD is atherosclerosis, an inflammatory disease developing in the arterial wall, promoted by dyslipidemia, diabetes mellitus, and hypertension^2–4^. As the number of deaths due to CAD is high, despite the existence of various treatments, there is a necessity to find new predictive biomarkers for CAD vulnerability and to develop new preventive therapies.

High-density lipoproteins (HDL) are macromolecular complexes of lipids and proteins, produced in the liver and small intestine, which transport lipids (free and esterified cholesterol, phospholipids and triglycerides) and proteins (apolipoproteins - apoAI, apoAII, apoCIII, apoE, enzymes) in the circulation^5–7^. In human plasma, HDL are represented by two major classes: HDL_2_ and HDL_3_, with different size, density and composition^6^. HDL have anti-atherosclerotic properties due to their potential to remove excess cholesterol from the peripheral tissues and transport it to the liver, the so called reverse cholesterol transport (RCT), but also for their anti-oxidant, anti-inflammatory and anti-thrombotic potential^8^. Epidemiologic data established that an increase of HDL-cholesterol (HDL-C) level is not enough for the improvement of clinical parameters in CAD patients^9^. A new hypothesis was proposed stating that HDL functionality is more relevant for the status of CAD patients compared to HDL-C levels^7, 10^. The procedures used to assess HDL functionality include the test of their potential to protect low density lipoproteins (LDL) from oxidation or to remove excess cholesterol from lipid-loaded macrophages^11^. In CAD patients, HDL dysfunction manifested as increased pro-apoptotic potential compared to healthy subjects. Little is known about the differences in HDL_2_ and HDL_3_ functionality between CAD patients with ACS and stable angina.

However, it is accepted that HDL functionality is closely related to the composition and quality of the molecules contained. Apolipoprotein AI (apoAI) is the main protein of HDL and its integrity is known to be very important for HDL function. The second most abundant apolipoprotein in HDL is apoAII, but its role in human HDL metabolism is still unclear^12^. ApoCIII is a minor component of HDL, being more associated with triglyceride (TG)-rich lipoproteins (TRL) which are independent risk factors for CAD development^13^. ApoC-III delays the lipolysis of TG and the catabolism of TRL remnants, thus raising plasma TG levels. Paraoxonase 1 (PON1) is an antioxidant enzyme physically associated with HDL particles, its activity being dependent on the levels of apoAI, apoAII and myeloperoxidase (MPO) in HDL^6, 7^. PON1 is a Ca^2+^-dependent serum esterase that is synthesized mainly in the liver, but also in the intestine and kidney^14–16^. The enzyme has aryl esterase, paraoxonase and lactonase activities, being able to degrade oxidized phospholipids and hydrolyze lactones from lipoproteins and cells^14^. HDL-associated PON1 plays an important role in protecting LDL against oxidation^17–19^. Data from literature demonstrate that the antioxidant capacity of HDL increases with PON1 content of HDL_3_^20, 21^. Recent studies have revealed that PON1 activity in sera and HDL of CAD patients is lower compared to healthy subjects, and concluded that HDL becomes oxidized and consequently dysfunctional^22–25^.

Ceruloplasmin (CP) is an acute phase protein that normally carries about 95% of the plasma copper. CP is considered an independent risk factor for atherosclerosis, CAD patients having an elevated serum CP^26^. CP can exhibit pro-oxidant activity, dependent on its structural integrity^27^. Previously, Navab *et al*. showed that during an acute phase (AP) response, AP-HDL from human patients, but not normal HDL, exhibited increases in CP. This increase in CP was not detected in acute phase VLDL or LDL^28^.

In this study, we aimed to identify the individual contribution of HDL subclasses to HDL dysfunction, the alterations in the composition of HDL subfractions (proteins, enzymes’ activity and oxidative status) that make them dysfunctional and estimate whether these modifications can discriminate between ACS and stable CAD patients, these differences between ACS and SA being at the moment unknown. To validate the data regarding the altered composition of HDL subclasses, we have evaluated *in vitro* their anti-inflammatory potential in TNF-α-activated human endothelial cells (EC).

## Results

### Subjects

The clinical characteristics (anthropometric and clinical data, medication) and plasma biochemical parameters of the CAD patients enrolled in the study are summarized in Tables 1 and 2. The subjects included in the study have ages with a mean of 61.7 ± 8.0 for stable angina (SA), 62.9 ± 9.1 for acute coronary syndrome (ACS), and 41.8 ± 9.5 for healthy (N) subjects. A significant increase in the body mass index (BMI) and glucose concentrations in the sera of all CAD patients (15%, p = 0.0003 and 16%, p = 0.048 respectively, for SA, and 12%, p = 0.003 and 21%, p = 0.021 respectively, for ACS) compared to N subjects was found (Table 2).

**Table 1:** Clinical characteristics of CAD patients and healthy subjects.

Parameter	N (n = 28)	SA (n = 40)	ACS (n = 53)
**Anthropometric data**			
Age (years)	41 ± 9.5	61 ± 8.0^***^	63 ± 9.0^***^
Gender Male (n, %)	12 (43%)	26 (65%)^***^	31 (58%)^***^
BMI (kg/m^2^)	25 ± 0.8	30 ± 0.9^***^	28 ± 0.7^**^
**Diagnosis**			
Obese (BMI > 30 kg/m^2^) (n, %)	4 (14%)	17 (42%)	23 (42%)
Diabetes mellitus (n, %)	0 (0%)	15 (37%)	19 (35%)
Hypertension (n, %)	0 (0%)	32 (80%)	38 (71%)
**Treatment**			
Statin therapy (n, %)	0 (0%)	40 (100%)	53 (100%)
Aspirin (n, %)	0 (0%)	30 (75%)	36 (67%)
Anti-platelets therapy (n, %)	0 (0%)	20 (50%)	30 (56%)
Anti-diabetic therapy (n, %)	0 (0%)	15 (37%)	19 (35%)
ACE-Inhibitor (n, %)	0 (0%)	29 (72%)	38 (71%)

N - healthy subjects, SA - stable angina patients and ACS - acute coronary syndrome patients; n - number of subjects, BMI - body mass index.

Data for age are expressed as means ± standard deviation (SD). The BMI data are expressed as means ± standard error of the mean (SEM) and analyzed with two-tailed Oneway ANOVA with Least Significant Difference (LSD) Post-hoc test. Chi-squared (χ^2^) analysis was used to evaluate the differences between two groups of binary logistic data (gender, obesity, diabetes, hypertension, medication). **p < 0.01, ***p < 0.001 vs. N.

**Table 2:** Plasma biochemical data from CAD patients and healthy subjects.

Parameter	N (n = 28)	SA (n = 40)	ACS (n = 53)
C (mmol/L)	5.20 ± 0.16	4.00 ± 0.15^***^	4.80 ± 0.15^###^
TG (mmol/L)	0.97 ± 0.07	1.60 ± 0.14^***^	1.92 ± 0.14^***,#^
Glucose (mmol/L)	5.50 ± 0.14	6.50 ± 0.43^*^	7.00 ± 0.46^*^
LDL-C (mmol/L)	3.25 ± 0.13	2.12 ± 0.12^***^	3.07 ± 0.15^###^
HDL-C (mmol/L)	1.57 ± 0.06	1.13 ± 0.05^***^	1.08 ± 0.05^***^
ApoAI (mg/dl)	131.10 ± 5.36	110.05 ± 5.13^***^	113.09 ± 4.90^**^
ApoE (mg/dl)	1.63 ± 0.09	2.42 ± 0.21^*^	3.18 ± 0.35^***^
CETP activity (μmol/L/h)	74.00 ± 4.40	64.10 ± 3.30	70.05 ± 3.20

N - healthy subjects, SA - stable angina patients, and ACS - acute coronary syndrome patients; n - number of subjects, C - total cholesterol, TG - triglycerides, LDL-C – low-density lipoproteins cholesterol, HDL-C – high-density lipoproteins cholesterol, ApoAI - apolipoprotein AI, ApoE –apolipoprotein E; CETP - cholesteryl esters transfer protein.

Data are expressed as means ± SEM and analyzed with two-tailed Oneway ANOVA with Least Significant Difference (LSD) Post-hoc test. *p < 0.05, **p < 0.01, ***p < 0.001 vs. N; ^#^p < 0.05, ^###^p < 0.001 vs. SA.

### Plasma biochemical parameters

**Figure 1:**
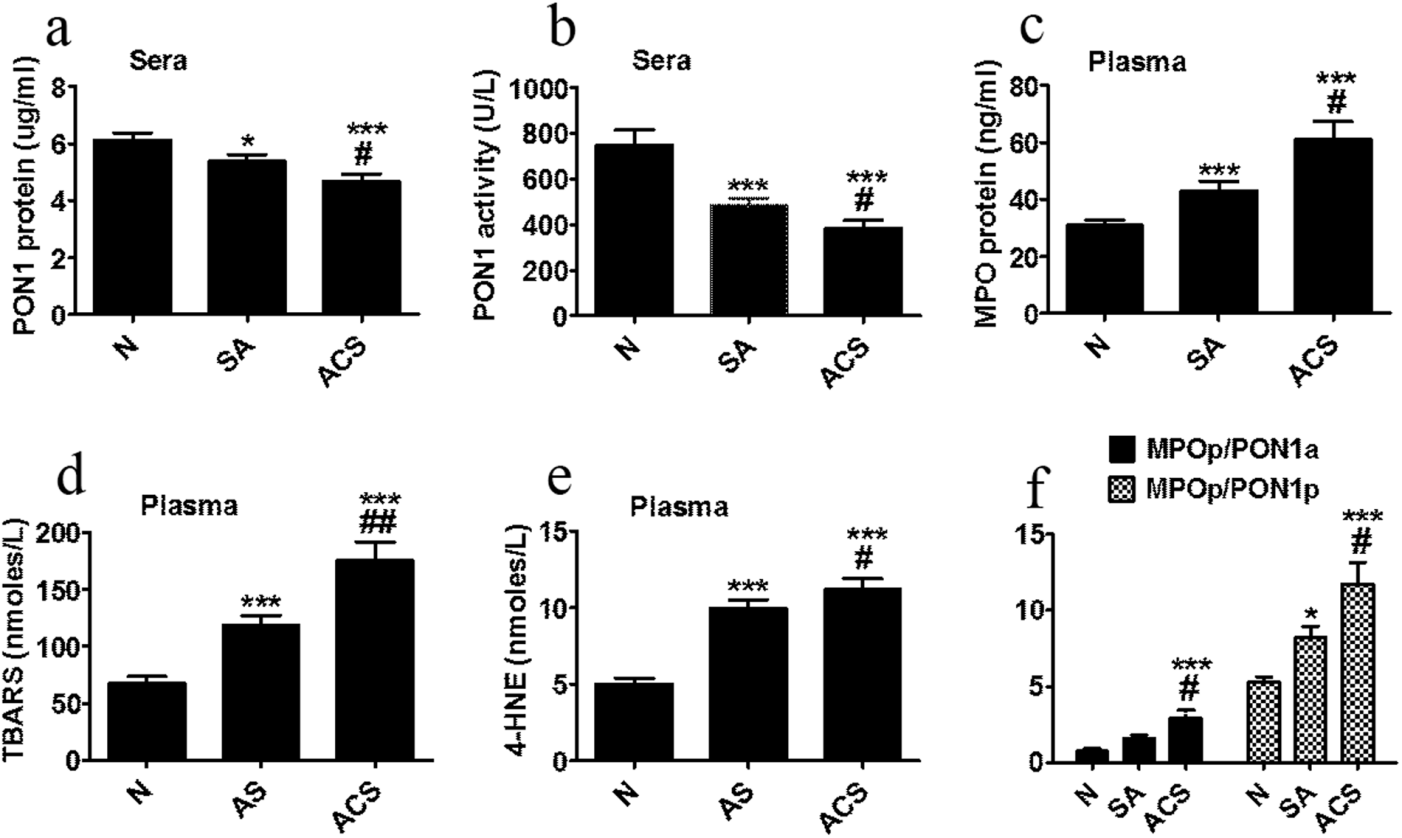
Biochemical parameters measured in sera/plasma of healthy subjects (N), stable angina patients (SA) and acute coronary syndrome patients (ACS). Levels of serum paraoxonase 1 (PON1) protein (**a**) and paraoxonase activity (**b**), plasma myeloperoxidase (MPO) protein (**c**), thiobarbituric reactive substances (TBARS) (**d**), free 4-hydroxy-2-nonenal (4-HNE) (**e**), and MPO protein/PON1 activity (MPOp/PON1a) and MPO protein/PON1 protein (MPOp/PON1p) ratios (**f**). Data are expressed as means ± SEM and analyzed with two-tailed Oneway ANOVA with Least Significant Difference (LSD) Post-hoc test; *p < 0.05, ***p < 0.001 vs. N; ^#^p < 0.05, ^##^p < 0.01 vs SA.

Plasma cholesterol (C) levels were significantly decreased in SA patients compared to N subjects (23%, p = 1.05 × 10^−6^), probably due to the intensive treatment with statins, but they were significantly higher in ACS (20%, p = 0.0002) compared to SA plasma (Table 2). TG concentrations were increased in SA (65%, p = 0.0007) and ACS (96%, p = 1.3 × 10^−5^) compared to N, and in ACS (19%, p = 0.048) versus SA patients plasma (Table 2). LDL-cholesterol (LDL-C) levels were significantly lower in SA patients compared to N subjects (35%, p = 2.6 × 10^−8^), but they were higher in ACS (41%, p = 3.5 × 10^−6^) compared to SA (Table 2). HDL-C levels in all CAD patients were significantly lower compared to N subjects (28%, p = 0.0001 for SA, and 31%, p = 1.25 × 10^−5^ for ACS) (Table 2). ApoAI concentrations were significantly lower in plasma of all CAD patients compared to N subjects (16%, p = 0.0065 for SA, and 14%, p = 0.008 for ACS) (Table 2). The apoE levels were increased in all CAD patients (48%, p = 0.046 for SA and 95%, p = 0.0002 for ACS) compared to N subjects (Table 2). The cholesteryl esters transfer protein (CETP) activity did not differ between groups (Table 2). PON1 protein levels in patients’ sera followed the same trend as HDL-C and apoAI levels, with a statistically significant decrease in all CAD patients versus N subjects (SA, 12%, p = 0.047; ACS, 23%, p = 0.0004), and also in ACS compared to SA (13%, p = 0.049) (Fig. 1a). PON1 activity in sera was decreased in all CAD patients compared to N subjects (SA, 35%, p = 0.0003; ACS, 48%, p = 4.8 × 10^−7^) and was significantly lower in ACS (20%, p = 0.04) compared to SA patients sera (Fig. 1b).

In addition, we analyzed PON1 activity in connection with HDL-C and apoAI levels in the sera of CAD patients and N subjects, and found a positive correlation between HDL-C or apoAI levels and PON1 activity (r = 0.273, p = 0.004 and r = 0.221, p = 0.013, respectively) (Figure S1a, S1b). No correlation was found between PON1 protein and HDL-C or apoAI levels.

We evaluated the levels of oxidative stress markers in plasma, such as: the pro-oxidant enzyme MPO, the thiobarbituric acid reactive substances (TBARS), free 4-hydroxy-2-nonenal (4-HNE) and oxidized LDL (oxLDL). The MPO concentrations in plasma of CAD patients were higher than in N subjects (SA, 38%, p = 0.0009; ACS, 96%, p = 3.9 × 10^−5^), and in ACS versus SA plasma (41%, p = 0.042) (Fig. 1c). TBARS levels were higher in all CAD patients’ compared to N subjects plasma (SA, 76%, p = 0.00003; ACS, 160%, p = 6.2 × 10^−7^), and also in ACS versus SA plasma (47%, p = 0.008) (Fig. 1d). Similarly, the free 4-HNE levels doubled in the plasma of all CAD patients versus N subjects (p = 0.0003 for SA and p = 1.2 × 10^−5^ for ACS). Furthermore, 4-HNE levels were significantly increased in ACS (12%, p = 0.038) compared to SA plasma (Fig. 1e). The oxLDL levels were significantly augmented in ACS compared to SA patients (23%, p = 0.034) and N subjects (38%, p = 0.023) (Figure S2). The ratio between MPO protein and PON1 activity was significantly higher in ACS versus SA and N (85%, p = 0.014, and 280%, p = 0.0008, respectively); the ratio of MPO protein to PON1 protein was also increased in CAD patients versus N subjects (SA, 56%, p = 0.012, and ACS, 120%, p = 0.00016), and in ACS versus SA (42%, p = 0.023) (Fig. 1f).

We performed a binary logistic regression (BLR) analysis to assess the relationship between the oxidative markers and HDL parameters in the cohort under study in order to find an approach to discriminate between SA and ACS patients. The BLR model showed that a panel of parameters comprising PON1 activity and mass protein, TBARS, MPO protein and apoAI levels, adjusted for age and gender, could significantly distinguish between ACS and SA patients, with an accuracy of over 82% (p = 0.034, Table S1). The highest contribution to this BLR model was brought by the ratio MPO protein/PON1 activity (odd ratio expB = 1723.33, Table S2), in good correlation with the increased oxidative status in plasma and HDL proteins of ACS versus SA patients. However, the BLR model including individually PON1 activity, TBARS or MPO levels, with no other covariates added, did not provide a statistically significant differentiation between the ACS and SA groups. The BLR model including PON1 and TBARS levels without any adjustment for age or gender as covariates resulted in a significant delineation between ACS versus SA patients with a moderate accuracy of around 68% (p = 0.026).

**Figure 2:**
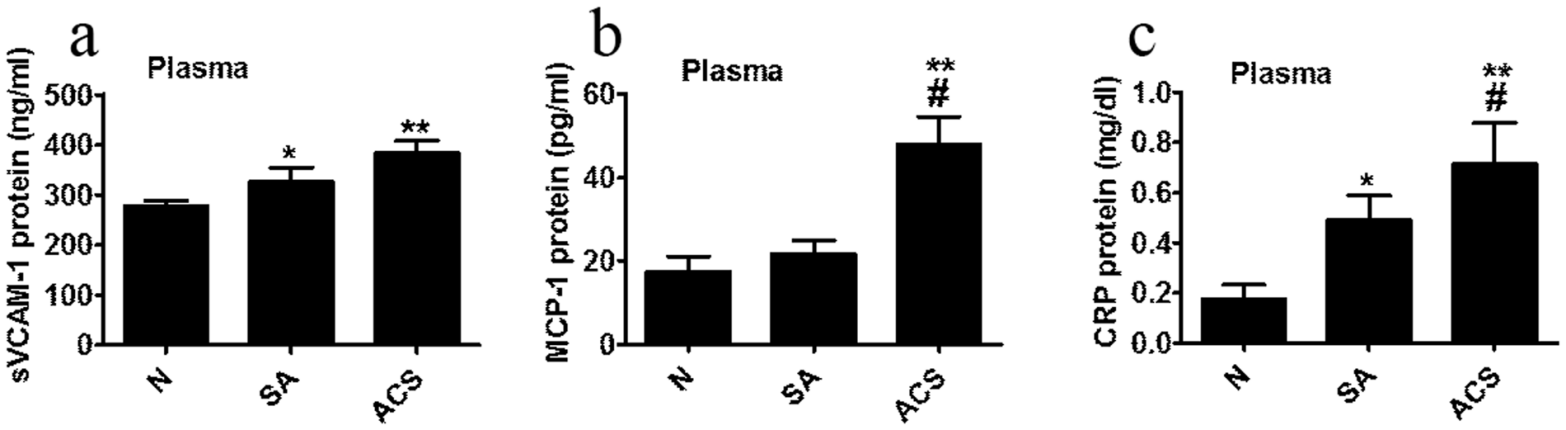
Inflammatory parameters measured in plasma of healthy subjects (N), stable angina patients (SA) and acute coronary syndrome patients (ACS). Levels of soluble vascular cell adhesion molecule 1 (sVCAM-1) (**a**), monocyte chemoattractant protein 1 (MCP-1) (**b**) and C reactive protein (CRP) (**c**). Data are expressed as means ± SEM and analyzed with two-tailed Oneway ANOVA with Least Significant Difference (LSD) Post-hoc test; *p < 0.05, **p < 0.01 vs. N; ^#^p < 0.05 vs. SA.

We also assessed some relevant pro-inflammatory molecules present in the plasma of CAD patients and N subjects. The soluble vascular cell adhesion molecule 1 (sVCAM-1) concentration was increased in all CAD patients’ compared to N subjects (SA, 17%, p = 0.041; ACS, 38%, p = 0.0072) (Fig. 2a). Monocyte chemoattractant protein 1 (MCP-1) levels were higher in ACS compared to SA and N subjects (120%, p = 0.026, and 170%, p = 0.0063 respectively) (Fig. 2b). The C reactive protein (CRP) levels were considerably augmented in CAD patients versus N subjects (SA, 180%, p = 0.014; ACS, 300%, p = 0.0027), and were significantly elevated in ACS compared to SA (45%, p = 0.038) (Fig. 2c).

### Characterization of HDL subfractions isolated from the plasma of ACS and SA patients

HDL_2_ and HDL_3_ subfractions separated by ultracentrifugation were collected based on the density of the fractions, the protein and cholesterol profiles (Figure S3). We performed seven ultracentrifugation isolations using between 6–8 patients/pool of plasma, and a total of 40 SA and 53 ACS patients. Characterization of HDL_2_ and HDL_3_ was performed by measuring the size of the particles, apolipoproteins (apoAI, apoAII, apoCIII, apoE and CP) and lipid content (C, TG, and phospholipids-PL). The results showed no statistically significant differences in the size, lipid content and apoE levels of HDL_2_ or HDL_3_ between the studied groups (Table 3).

**Table 3:** Characteristic parameters of HDL_2_ and HDL_3_ subfractions isolated from sera of normal subjects (N), stable angina patients (SA) and acute coronary syndrome (ACS) patients.

	HDL_2_			HDL_3_		
	N	SA	ACS	N	SA	ACS
Density (g/L)	1.114			1.139		
Particle size (nm)	9.50 ± 0.41	9.36 ± 0.69	9.82 ± 0.20	8.40 ± 0.20	8.60 ± 0.20	8.57 ± 0.40
Protein (µg/ml)	560.90 ± 165.00	556.80 ± 190.32	506.90 ± 185.09	1109.01 ± 520.05	1150.00 ± 320.12	1354.15 ± 486.17
C (µg/ml)	179.85 ± 73.01	165.11 ± 55.01	140.20 ± 43.92	127.17 ± 52.00	130.23 ± 60.02	125.00 ± 62.03
TG (µg/ml)	38.51 ± 9.35	34.10 ± 9.20	39.62 ± 17.02	30.10 ± 10.06	28.40 ± 6.01	34.25 ± 9.55
PL (µg/ml)	29.60 ± 12.8	25.32 ± 7.79	24.30 ± 7.20	21.10 ± 9.30	22.40 ± 7.90	24.82 ± 8.96
ApoE (µg/ml)	1.07 ± 0.41	0.87 ± 0.30	1.20 ± 0.42	2.20 ± 0.60	1.71 ± 0.22	2.01 ± 0.20

N - healthy normal subjects, SA - stable angina patients, and ACS - acute coronary syndrome patients; C - cholesterol, TG - triglycerides, PL - phospholipids, ApoE - apolipoprotein E.

Data are expressed as means ± SD and analyzed with Independent Student’s T-test (n = 7).

To further investigate if differences exist between HDL subfractions, we searched for other parameters that characterize these lipoproteins in CAD patients.

#### HDL_2_ parameters

**Figure 3:**
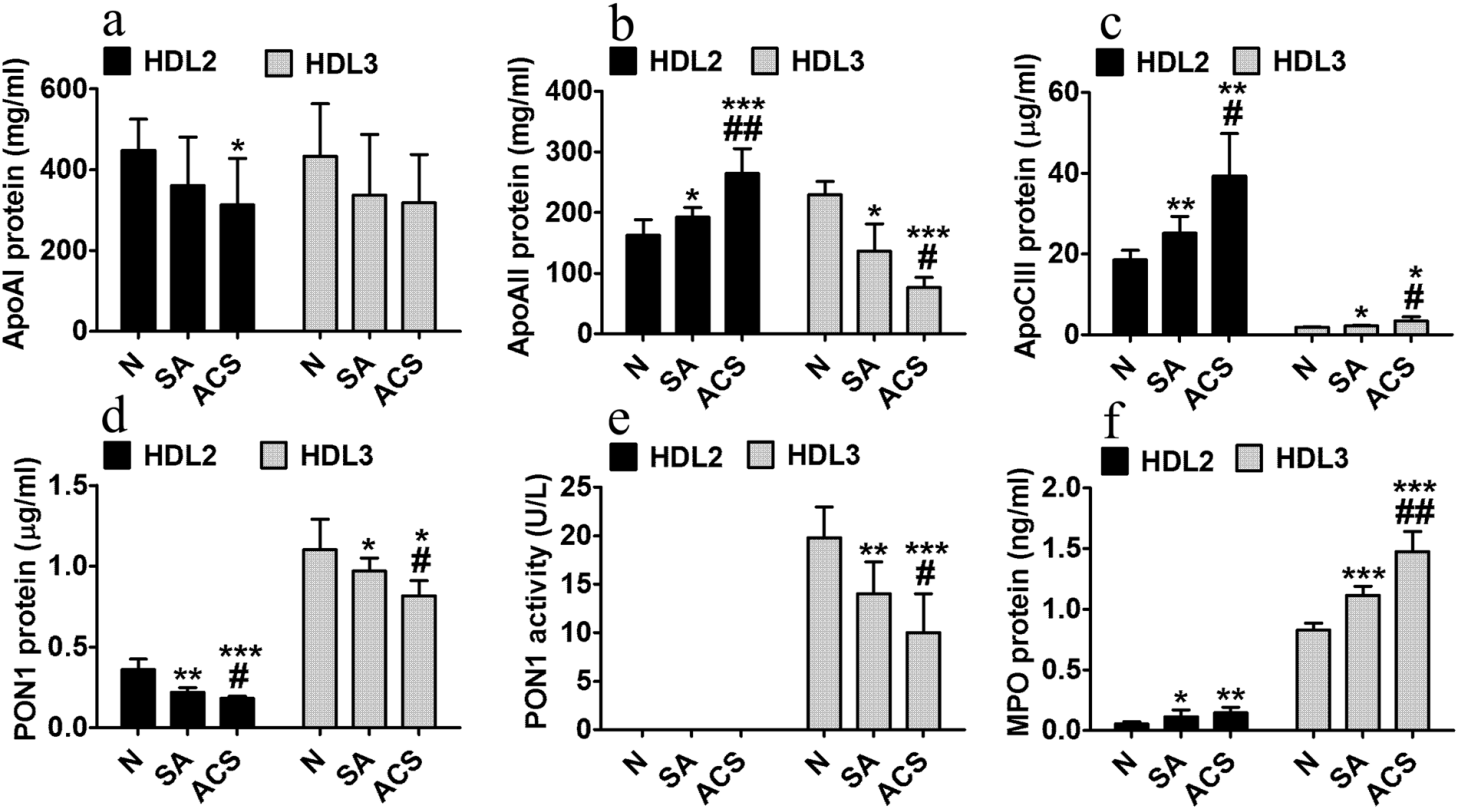
Characterization of HDL_2_ and HDL_3_ subfractions isolated by density gradient ultracentrifugation from seven independent pools of sera collected from healthy subjects (N), stable angina patients (SA) and acute coronary syndrome (ACS) patients. Levels of apolipoprotein AI (ApoAI) (**a**), ApoAII (**b**), ApoCIII (**c**), paraoxonase 1 (PON1) protein (**d**) and paraoxonase activity (**e**), and myeloperoxidase (MPO) protein (**f**). Data are expressed as means ± SD and analyzed with Independent Student’s T-test; *p < 0.05, **p < 0.01, ***p < 0.001 vs. N, ^#^p < 0.05, ^##^p < 0.01 vs. SA.

**Figure 4:**
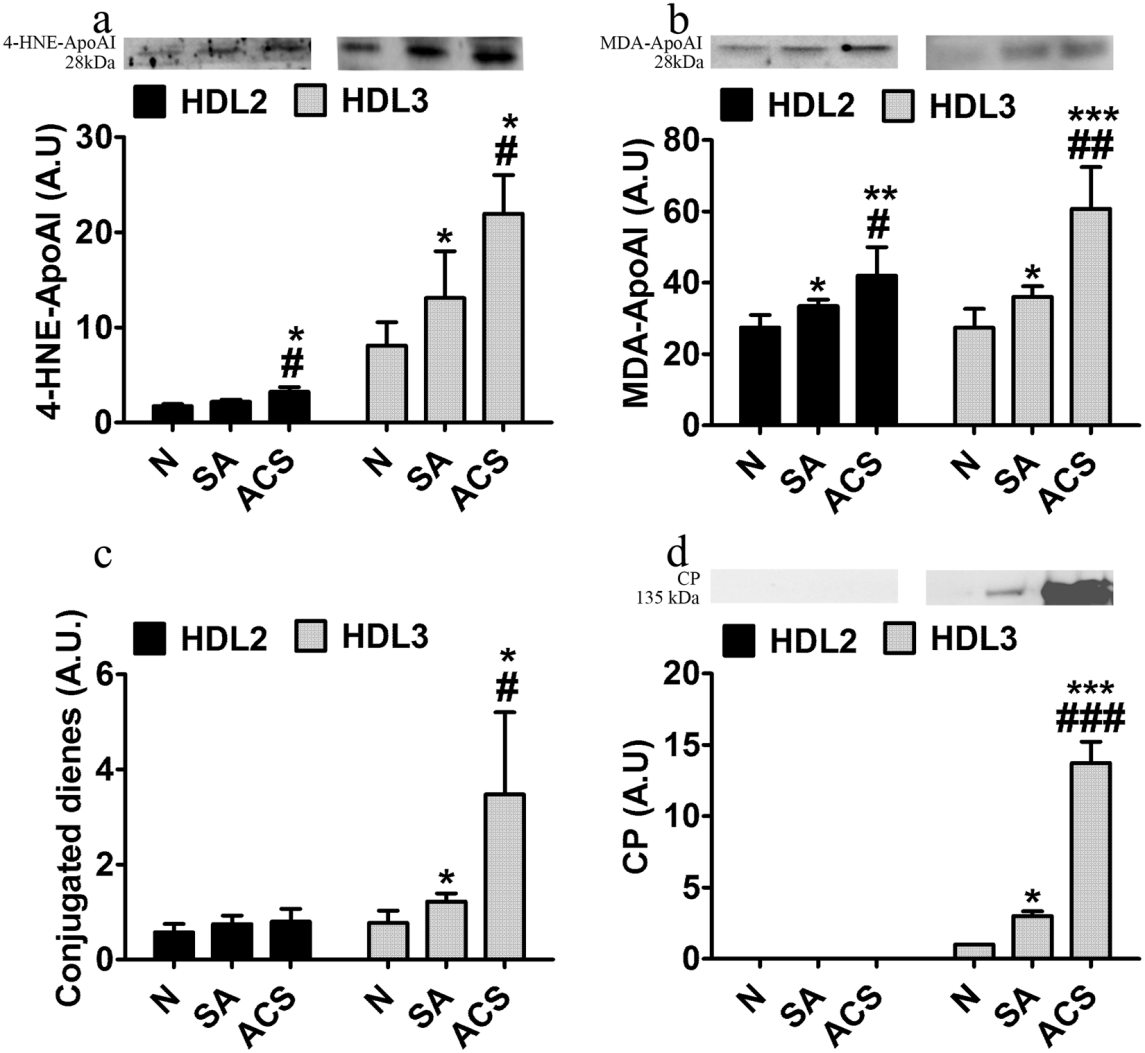
Characterization of oxidative markers in HDL_2_ and HDL_3_ subfractions isolated by density gradient ultracentrifugation from seven independent pools of sera from healthy subjects (N), stable angina patients (SA) and acute coronary syndrome (ACS) patients. Levels of 4-hydroxy-2-nonenal associated to apolipoprotein AI (4-HNE-apoAI) (**a**), malondialdehyde associated to apolipoprotein AI (MDA-apoAI) (**b**), conjugated dienes (**c**) and ceruloplasmin (CP) (**d**). The western blot images were processed by cropping the corresponding parts from the blots. The full-length blots are supplied in the Supplementary information. Data are expressed as means ± SD and analyzed with Independent Student’s T-test; *p < 0.05, **p < 0.01, ***p < 0.001 vs. N, ^#^p < 0.05, ^##^p < 0.01 vs. SA.

ApoAI levels did not differ between the CAD groups, but they were statistically decreased in ACS versus N (27%, p = 0.02) (Fig. 3a). The levels of apoAII were significantly higher in HDL_2_ from SA and ACS compared to N subjects (18%, p = 0.047 and 63%, p = 0.0008, respectively), and in ACS compared to SA (37%, p = 0.006) (Fig. 3b). ApoCIII levels were significantly elevated in HDL_2_ from SA and ACS compared to N subjects (32%, p = 0.007 and 111%, p = 0.006 respectively), and in ACS versus SA (56%, p = 0.045) (Fig. 3c). PON1 protein levels in HDL_2_ were significantly decreased in SA and ACS compared to N (38%, p = 0.006 and 50%, p = 0.0009, respectively), and in ACS versus SA (18%, p = 0.048) (Fig. 3d). PON1 activity was under the detection limit in HDL_2_. MPO protein levels were significantly higher in HDL_2_ from ACS (167%, p = 0.009) and SA (103%, p = 0.021) compared to N (Fig. 3f). The levels of 4-HNE-apoAI were significantly increased in HDL_2_ from ACS compared to SA and N subjects (48%, p = 0.048 and 87%, p = 0.031 respectively) (Fig. 4a). The levels of MDA-apoAI were significantly increased in HDL_2_ from SA and ACS compared to N subjects (21%, p = 0.019 and 55%, p = 0.005, respectively), and in ACS versus SA patients (27%, p = 0.043) (Fig. 4b). The conjugated dienes showed no statistically significant differences between groups (Fig. 4c). No CP was detected in HDL_2_ subfraction by Western blot (Fig. 4d).

In summary, HDL_2_ from ACS versus SA patients have statistically significant higher levels of apoAII, apoCIII, MPO protein, 4-HNE-apoAI, MDA-apoAI and very low PON1 protein.

#### HDL_3_ parameters

ApoAI levels did not differ significantly between HDL_3_ from CAD groups, but tended to decrease in SA and ACS compared to N subjects (Fig. 3a). The levels of apoAII were significantly lower in HDL_3_ from SA and ACS compared to N subjects (40%, p = 0.032 and 66%, p = 0.0006, respectively), and in ACS versus SA (44%, p = 0.048) (Fig. 3b). Conversely, the levels of apoCIII were increased in HDL_3_ from SA and ACS compared to N subjects (16%, p = 0.048 and 83%, p = 0.026, respectively), and in ACS versus SA (57%, p = 0.047) (Fig. 3c). PON1 protein levels were lower in HDL_3_ from ACS compared to SA and N subjects (16%, p = 0.047 and 27%, p = 0.03, respectively) (Fig. 3d). In parallel, PON1 activity in HDL_3_ was significantly decreased in all CAD patients (SA, 30%, p = 0.005; ACS, 50%, p = 0.0003) compared to N subjects (Fig. 3e). PON1 activity was significantly reduced in HDL_3_ from ACS compared to SA patients (28%, p = 0.041), a deeper decrease than the one of PON1 protein (Fig. 3e). MPO levels were significantly higher in SA and ACS compared to N (35%, p = 0.0009 and 79%, p = 0.0003, respectively), and in ACS compared to SA (32%, p = 0.007) (Fig. 3f). The levels of 4-HNE-apoAI were significantly increased in HDL_3_ from SA and ACS compared to N subjects (60%, p = 0.038 and 170%, p = 0.018, respectively), and in ACS versus SA (68%, p = 0.047) (Fig. 4a). The levels of MDA-apoAI were significantly heightened in HDL_3_ from SA and ACS compared to N subjects (31%, p = 0.017 and 122%, p = 0.0003, respectively), and from ACS compared to SA (69%, p = 0.0018) (Fig. 4b). The conjugated dienes were augmented in HDL_3_ from SA versus N (58%, p = 0.046), and more increased in ACS versus SA (184%, p = 0.032) (Fig. 4c). CP levels differed dramatically between HDL_3_ from CAD groups, having a huge value in ACS compared to SA, and almost zero level in N subjects (Fig. 4d).

In summary, HDL_3_ from ACS versus SA patients have statistically significant higher levels of apoCIII, MPO protein, conjugated dienes, 4-HNE-apoAI, MDA-apoAI and lower levels of apoAII, PON1 protein and activity.

### The anti-inflammatory effect of HDL subfractions on TNFα-activated EC

We aimed at the evaluation of the anti-inflammatory potential of HDL subfractions by co-incubating TNFα-activated EC with HDL_2_ or HDL_3_, followed by the assessment of the synthesis and secretion of several well-known pro-inflammatory molecules, VCAM-1, MCP-1 and CRP.

**Figure 5:**
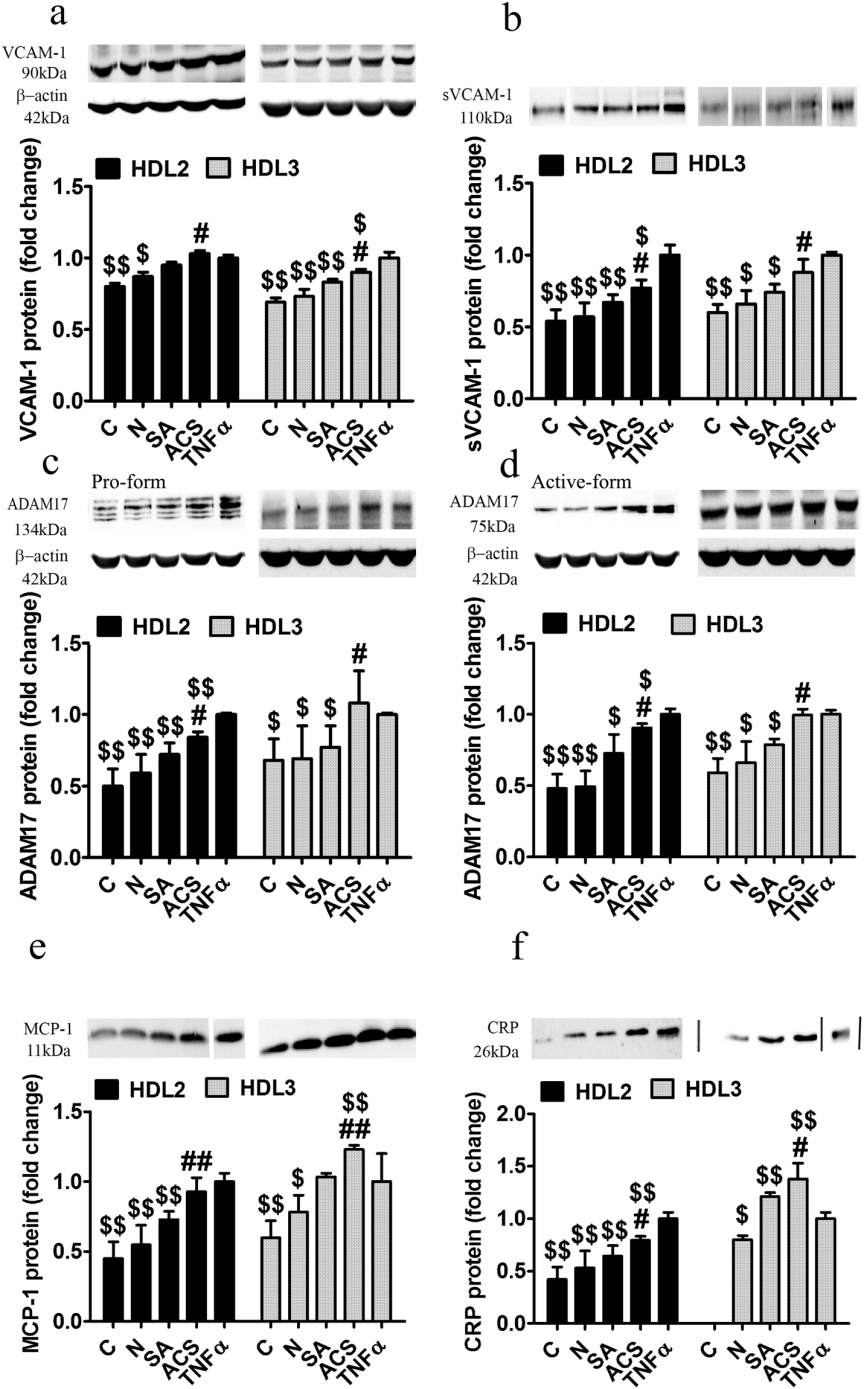
HDL_2_ and HDL_3_ functionality evaluated by their anti-inflammatory effects on endothelial cells (EC) activated with tumor necrosis factor α (TNFα). The vascular cell adhesion molecule 1 (VCAM-1) protein relative to β-actin (**a**) and soluble VCAM-1 (sVCAM-1) in the cultured media relative to cellular protein (**b**); protein expression of disintegrin and metalloproteinase domain-containing protein 17 (ADAM17) pro-form (**c**) and active form (**d**) relative to β-actin. Levels of monocyte chemoattractant protein 1 (MCP-1) (**e**) and C reactive protein (CRP) (**f**) secreted in the culture medium relative to cellular protein. All data come from four different experiments and are expressed as fold change versus TNFα-activated EC. C - cells unstimulated with TNFα, N - healthy subjects, SA - stable angina patients, ACS - acute coronary syndrome patients, TNFα - tumor necrosis factor α. The western blot (wb) images for (**b**), (**e**) and (**f**) were processed by cropping the lanes from the same blot and marked by dividing white lines where the order of the samples on wb was different from the one in the graph. The full-length blots are supplied in the Supplementary information. Data are presented as means ± SD and analyzed with Independent Student’s T-test. ^#^p < 0.05, ^##^p < 0.01 vs. SA, ^$^p < 0.05, ^$$^p < 0.01 vs. TNFα.

HDL_2_ from N subjects induced a 15% (p = 0.014) decrease of VCAM-1 protein expression compared to TNFα-exposed EC (Fig. 5a). HDL_2_ from either of the two CAD groups exhibited no protective effect on activated EC in terms of cellular protein expression of VCAM-1; however, VCAM-1 levels were significantly higher in the culture media of HDL_2_ from ACS compared to SA (10%, p = 0.044) (Fig. 5a). The levels of soluble VCAM-1 (sVCAM-1) secreted by TNFα-exposed EC were significantly reduced by HDL_2_ from SA (33%, p = 0.004) and to a lesser extent by HDL_2_ from ACS (23%, p = 0.016), the difference between the effect of HDL_2_ from ACS and SA being statistically significant (15%, p = 0.043) (Fig. 5b).

HDL_3_ from both CAD groups induced a diminished VCAM-1 protein expression in activated EC (17%, p = 0.0035 for SA; 10%, p = 0.034 for ACS), and a 9% reduction in SA versus ACS (p = 0.041) (Fig. 5a). HDL_3_ from ACS induced no significant decrease of sVCAM-1 in the culture medium of activated EC compared to HDL_3_ from SA (26%, p = 0.026) (Fig. 5b).

It was reported that the active form of disintegrin and metalloprotease domain 17 (ADAM17) is responsible for cleaving VCAM-1 from the EC membrane to give sVCAM-1 in the culture medium^29^. We found that ADAM17 protein levels were significantly reduced in HDL_2_ from ACS (10%, p = 0.046) and SA (27%, p = 0.02) compared to TNFα-activated EC (Fig. 5d). The determined increased levels of sVCAM-1 in the case of HDL_2_ from CAD compared to N subjects correlated with the increased levels of ADAM17 protein. ADAM17 protein levels were significantly increased in HDL_2_ from ACS compared to SA (20%, p = 0.048), in good correlation with the higher levels sVCAM-1 (Fig. 5d). HDL_3_ from SA induced a significant decrease compared to TNFα-exposed EC (20%, p = 0.038), while the HDL_3_ from ACS patients had no effect on ADAM17 protein expression, that is in accordance with the sVCAM-1 protein levels in the culture medium (Fig. 5d). The protein expression of ADAM17 pro-form followed the same trend as the active form (Fig. 5c).

Co-incubation of EC with HDL_2_ from SA patients induced a 27% (p = 0.003) decrease of the secreted MCP-1 level compared to HDL_2_ from N subjects (45%, p = 0.0015) both figures expressed relative to TNFα-activated EC. In contrast, HDL_2_ from ACS induced only a modest, statistically insignificant decrease of the secreted MCP-1 level (Fig. 5e). HDL_3_ from SA had no protective effect on activated EC in terms of MCP-1 secretion, while HDL_3_ from ACS exhibited a significant pro-inflammatory effect by inducing a 23% (p = 0.0023) increase of the secreted MCP-1 by activated EC. As expected, HDL_3_ from N subjects induced a significant reduced secretion of MCP-1 (22%, p = 0.022) (Fig. 5e).

Incubation of TNFα-activated EC with HDL_2_ from ACS induced a 20% (p = 0.0015) decrease of the secreted CRP level, which was significantly smaller (23%, p = 0.016) compared to the effect of HDL_2_ from SA (36%, p = 0.0055) versus activated EC (Fig. 5f). HDL_3_ from both CAD groups exhibited pro-inflammatory effects in TNFα-exposed EC, inducing a 21% (p = 0.0078) increase of secreted CRP levels for SA and 38% (p = 0.0057) for ACS, the 14% increase for ACS versus SA being statistically significant (p = 0.042) (Fig. 5f).

In summary, the anti-inflammatory potential of HDL_2_ from ACS versus SA in activated EC determined significantly higher levels of sVCAM-1, ADAM-17, MCP-1 and CRP, while HDL_3_ induced a pro-inflammatory effect in the case of all measured inflammatory molecules.

## Discussions

In physiological conditions, HDL have anti-atherosclerotic, anti-inflammatory and anti-thrombotic properties^8^. Our hypothesis was that in CAD patients modifications of the apolipoproteins and enzymes in HDL are associated with progressive functional alterations that could explain the differences between ACS and SA patients. Together with other known biomarkers, these changes in HDL composition could be further used for a better prediction of the major adverse cardiac events (MACE) in CAD patients.

The novel findings of our study are: (i) HDL_2_ and HDL_3_ from CAD patients are dysfunctional as revealed by the increased apoCIII, MPO and oxidatively-modified apoAI levels; these alterations are more pronounced in HDL_3_ from ACS than from SA patients; (ii) apoAII levels are increased in HDL_2_ (ACS > SA), in contrast with their decrease in HDL_3_ (ACS < SA); (iii) PON1 protein and activity are decreased, and CP is increased in HDL_3_ from ACS versus SA patients; (iv) the anti-inflammatory effect of HDL_2_ and HDL_3_ assessed on the activated EC is lower for ACS versus SA, as reflected by the increase in ADAM17-mediated sVCAM-1, MCP-1 and CRP levels. These modifications were detected despite of the extensive medication that substantially ameliorated some of the other plasma biochemical parameters, such as total cholesterol and LDL-C.

The concept of dysfunctional HDL emerged from the observations that CAD patients with normal HDL-C, but low PON1 activity and increased lipid peroxides in plasma and in HDL, compared to healthy subjects, have decreased plasma anti-oxidant potential that favors the oxLDL-induced inflammation^10^. The anti-inflammatory and antioxidant potentials of HDL are closely related and appear to depend on complex, not yet deciphered, interactions between their apolipoproteins and enzymes.

It is accepted that oxidized apoAI is a marker of dysfunctional HDL^30^, and our data show for the first time that MDA-apoAI and 4-HNE-apoAI levels are significantly increased in HDL_2_ and HDL_3_ (HDL_3_ > HDL_2_) from ACS compared to SA. The oxidative modifications of HDL subfractions are additionally supported by the increased level of conjugated dienes in ACS compared to SA, particularly in HDL_3_. MDA-apoAI appearance could be due to the increase of MPO/PON1 ratio in HDL_2_ and HDL_3_ (HDL_3_ > HDL_2_) from ACS compared to SA, in accordance with the TBARS and MPO levels measured in plasma (ACS > SA)^30^. Here we report for the first time that ceruloplasmin is firmly associated with HDL_3_ and very little with HDL_2_ from CAD patients and healthy subjects (ACS > SA > N). This could explain the increased oxLDL in plasma of ACS > SA, since we have previously demonstrated that ceruloplasmin has a significant oxidant potential against LDL at neutral pH^31^.

It is largely accepted that PON1 is one of the major proteins which determines the antioxidant potential of HDL and its reduced activity contributes to the impairment of HDL quality^32^. Several studies have shown that the concentration and activity of PON1 are decreased in CAD patients compared to healthy subjects^23, 33^. These results are confirmed and extended by the present study in which we report that the significant decrease of PON1 activity in CAD patients’ sera is characteristically more prominent in ACS than SA. Our data are in contrast with the study of Yunoki *et al*. conducted on patients with stable and unstable angina showing that PON1 activity and protein do not differ between these two groups^34^. Our statistical analysis evidenced a significant positive correlation between PON1 activity, HDL-C and apoAI levels in sera of the studied groups that is in good agreement with other recent reports^33, 35^. Our data show for the first time that the levels of PON1 protein and activity are significantly lower in HDL_3_ from ACS < SA < N subjects. Moreover, the decrease of PON1 activity in HDL_3_ from ACS patients exceeded the decrease of its protein level suggesting the existence of dysfunctional PON1. The firm attachment of PON1 to HDL particles is mediated by its interaction with phospholipids and apoAI, and is influenced by the presence of MPO and apoAII^36^. It was reported that MPO may form a ternary complex with PON1 and HDL, and determine the oxidation of PON1, the ratio between these two enzymes in serum being proposed as marker for HDL functionality^37, 38^. Our data show that the ratio MPO protein/PON1 activity is increased by 85% in ACS versus SA sera, in good agreement with the former reports^37, 38^.

The role of apoAII in stabilizing the HDL structure and function is still controversial, being reported that it can impede PON1 binding to HDL^39^. Our data show for the first time that the levels of apoAII are increased in HDL_2_ from ACS versus SA patients, in contrast with HDL_3_, where apoAII levels are decreased in ACS versus SA. Thus, an explanation for the decreased levels of PON1 protein in HDL_2_ compared to HDL_3_ could be the increase of apoAII in CAD versus N subjects that favors PON1 displacement by MPO, which in turn oxidizes apoAI and weakens the PON1-apoAI link. Compared to SA, HDL_3_ from ACS has higher MPO levels in association with higher oxidized-apoAI levels compared to HDL_2_, which can explain the more reduced PON1 activity/PON1 protein ratio.

ApoCIII is a protein involved in HDL remodeling by lipases, known to increase plasma TG through inhibition of lipoprotein lipase^40^. There are no data about the relationship between apoCIII and MPO or PON1 in HDL. Our results show for the first time that apoCIII is increased in HDL_2_ and HDL_3_ (HDL_2_ > HDL_3_) from ACS compared to SA and N. The higher levels of apoCIII in HDL_2_ compared to HDL_3_ could be associated with the higher content of TG in HDL_2_ versus HDL_3_, as was previously reported^41^. It is known that functional HDL inhibit or attenuate the oxidation of LDL and of the membrane phospholipids^18, 42^. In accordance with the latter reports, our data show significant increases of oxLDL, TBARS, and free 4-HNE in ACS versus SA plasma. The presence of oxidative stress markers in higher amounts in ACS compared to SA supports the existence of dysfunctional HDL_2_ and HDL_3_, more prominent in ACS than in SA, despite of the intensive treatments of patients with statins, known to have anti-oxidant properties.

Atherosclerosis is an inflammatory disease and activation of the endothelium by various risk factors that trigger the EC to secrete pro-inflammatory molecules is among the most important processes in the inception and evolution of the atheroma^43^. It is accepted that the TNFα signaling is a central pro-inflammatory pathway involved in atherosclerosis progression^44^. To validate the functional significance of the demonstrated biochemical alterations of HDL, we assessed the anti-inflammatory potential of HDL_2_ and HDL_3_ from ACS and SA patients by measuring their effect on the inflammatory molecules secreted by TNFα-activated EC. We found that in ACS compared to SA, the levels of VCAM-1 protein expression and secretion in the presence of HDL_2_ and HDL_3_ were significantly higher, indicating a lower anti-inflammatory potential for both HDL subfractions. We show for the first time that the increased secretion of sVCAM-1 is due to a significantly increased expression of the metalloprotease ADAM17 in TNFα-exposed EC, and that HDL_2_ and HDL_3_ from ACS determine a smaller inhibition of ADAM17 protein expression than HDL from N subjects or SA patients. In addition, MCP-1 and CRP levels in the culture medium of TNFα-exposed EC incubated with HDL_2_ from ACS were increased compared to those from SA and N, strengthening the evidence for a more prominent dysfunction of HDL_2_ in ACS versus SA patients. HDL_3_ from ACS compared to SA induced higher levels of MCP-1 and CRP in the culture media of activated EC. These data suggest that HDL_3_ from the ACS group have pro-inflammatory action on EC, increasing the pro-inflammatory effect of TNFα. Our previous data and those from other laboratories show that VCAM-1 and MCP-1 expression is induced by activation of NF-kB in EC^45, 46^. It was reported that apoCIII per se is able to activate NF-kB in EC and increase the adhesion of monocytic cells^47^. Our results reveal an association between the increased apoCIII in HDL subfractions and the diminution of their anti-inflammatory properties. In good association with the *in vitro* data, we measured a significant increase of sVCAM-1, MCP-1 and CRP levels in the plasma of CAD patients (ACS > SA > N).

Taken together, our data show that HDL_3_ from CAD patients are more pro-oxidatively altered than HDL_2_ (increased oxidized-apoAI, MPO/PON1 and ceruloplasmin). Consequently, HDL_3_ from CAD patients are converted to pro-inflammatory lipoproteins, despite of the intensive statin treatment of the patients. These data are in agreement with our previous results showing that hyperlipidemia induces oxidation of PON1 and apoAI and their down-regulation in the small intestine and liver of hamsters, but the treatment with probiotics diminishes the oxidative stress^16, 48^. It is known that HDL play a key role in RCT and this function is affected by oxidative modifications, as was previously reported^49, 50^. Gibson C.M. *et al*. and Kallend D.G. *et al*. showed that the infusion of apoAI-based compound CS112 and MDCO-216 improved plasma cholesterol efflux, but did not reduce MACE and did not improve plaque regression^51, 52^. Taken together with our results, we can conclude that it is not only the level of apolipoprotein or enzyme, but also its quality that contributes to MACE.

In conclusion, the assessed panel of markers (apoCIII, MPO, oxidized-apoAI, ceruloplasmin, and PON1) correlates with the reduced anti-inflammatory potential of HDL_2_ and HDL_3_ (mostly HDL_3_ from ACS) isolated from ACS and SA patients and can discriminate between these groups of CAD patients. Approaches to develop new therapies for treatment of CAD patients at risk based on the stimulation of the liver and small intestine to produce functional HDL are the future challenge.

## Methods

### Reagents

All reagents used were from Sigma-Aldrich Co., MO, USA or Cayman Chemicals, MI, USA. Specific antibodies for each protein were used for Western blot (WB) analysis: rabbit polyclonal anti-soluble VCAM-1 (sc1504-R), goat polyclonal anti-4-hydroxynonenal-protein adducts (4-HNE) (sc-130083), and mouse monoclonal anti-β-actin were purchased from Santa-Cruz Biotechnology, TX, USA (sc47778). The mouse monoclonal anti-paraoxonase 1 (PON1) (ab24261), rabbit polyclonal anti-malondialdehyde-protein (ab27642), goat polyclonal anti-ceruloplasmin (ab19171), rabbit polyclonal anti-VCAM-1 (ab106777), rabbit polyclonal anti-MCP-1 (ab9669), mouse monoclonal anti-CRP (ab50861), rabbit polyclonal anti-ADAM17 (ab39162), and the secondary antibodies rabbit polyclonal anti-goat (ab6741-1), goat polyclonal anti-rabbit (ab6721), and rabbit anti-mouse (ab6728) labeled with horseradish peroxidase (HRP) were from Abcam, Cambridge, UK. The nitrocellulose membrane (0.4 μm or 0.22 μm) for WB was from BioRad Laboratories, Hercules, CA, USA. The Chemiluminescent HRP substrate (Immobilon Western) kit was from Millipore Corporation, Billerica, MA, USA.

### Subjects

Our study includes 121 subjects: 93 patients with CAD (36 women and 57 men, aged 35–79 years), and 28 controls (N) (16 women and 12 men, aged 25–62 years). The patients were split into 2 groups based on their clinical characteristics: (i) stable angina (SA, n = 40), and (ii) acute coronary syndrome, meaning unstable angina (ACS, n = 53). All patients were enrolled at the Cardiology Clinic, Elias Emergency University Hospital, Bucharest. The subjects of N group were healthy control donors, without a history of CAD risk factors or recurrent disease, from the Blood Transfusion Center, Bucharest. This study was carried out in accordance with the principles from the Declaration of Helsinki (The Code of Ethics of the World Medical Association, last updated at the 64^th^ WMA General Assembly, Fortaleza, Brazil, October 2013) for experiments involving humans. All participants gave their informed consent, respecting their privacy rights. The Ethics Committee of the Institute of Cellular Biology and Pathology “Nicolae Simionescu” has approved the study (authorization number 1382/17.09.2012).

### Blood samples

Blood samples were collected from each subject in fasted state. Sera and plasma were separated and stored at −80 °C for subsequent measurements.

### Lipoprotein isolation

For lipoproteins isolation, equal amounts of sera from patients of each group were randomly pooled; the procedure was performed on 7 independent pools of sera from each group (6–8 samples per pool). We performed isolation and characterization of HDL sub-fractions (HDL_2_ and HDL_3_) from the patients’ group-pooled sera as described^53^. Briefly, an aliquot of 1.25 mL pooled sera from each N, SA, and ACS groups was adjusted to a density of 1.23 g/mL with KBr (Sigma-Aldrich, St. Louis, MO, USA) and then overlaid with 2 mL of 1.21 g/mL KBr, 5 ml of 1.063 g/mL KBr, 1 mL of 1.019 g/mL KBr, and 1 mL of phosphate buffered saline (PBS). The mixture was ultracentrifuged for 18 h in a SW-41 Ti rotor at 30,000 rpm (154,000x g) in an Optima L-80XP ultracentrifuge (Beckman Coulter International SA, Nyon, Switzerland). Ten fractions (1 mL each) were collected from each tube and dialyzed against PBS, pH 7.4, at 4 °C in the dark. The fractions number 7–8 and 9 were of interest, corresponding to HDL_2_ (d = 1.06–1.12 g/ml) and HDL_3_ (d = 1.12–1.25 g/ml) subpopulations. The protein in the collected fractions was assessed by a modified Lowry method using bovine serum albumin as standard^54^.

### Serum parameters determination and HDL characterization

Total cholesterol (C) and triglycerides (TG) levels were determined employing automated biochemical analyzers. HDL cholesterol (HDL-C) and low density lipoproteins cholesterol (LDL-C) levels were measured by a commercially available kit (Dialab Gmbh., Neudorf, Austria). Phospholipids (PL) levels were determined by using a commercially available kit (Wako Chemicals GmbH, Neuss, Germany). ApoAI, apoAII, apoCIII, apoE, PON1, MPO, VCAM-1 and MCP-1 levels were determined by enzyme-linked immunosorbent assay kits commercially available (apoAI and apoE - Mabtech, Sweden, apoAII and apoCIII – Abnova Corporation, Taoyuan, Taiwan, PON1, MPO, VCAM-1 and MCP-1 - R&D Systems, Minneapolis, Minnesota, USA). CETP activity was determined by using a commercial available kit (Biovision Inc., CA, USA). The CRP levels were measured by a turbidimetric method using commercial available kit (Wako Chemicals GmbH, Neuss, Germany). PON1 activity was assessed by measuring the capacity of the enzyme to hydrolyze the paraoxon substrate using a method described by Rozenberg *et al*.^55^. Briefly, 10 µL serum samples prediluted (1:2) or undiluted HDL sub-fractions were added to 95 µL buffer solution (100 mM Tris-HCl, 2 mM CaCl_2_, pH 8.0) and then 95 µl of 5.5 mM paraoxon-ethyl (Sigma-Aldrich, Canada). PON1 activity was calculated based on the absorbance recorded at 412 nm during enzymatic reaction kinetic. Blanks without enzyme were used to correct for the spontaneous hydrolysis of the substrate. The dimension of the HDL particles were measured using the nanoparticle size analyzer (Agilent Nicomp-380), based on the dynamic light scattering principle. The density of lipoproteins fractions separated by density gradient ultracentrifugation was determined based of refraction indices measured with an Abbe refractometer.

### Determination of lipid oxidation products in plasma and HDL

The levels of thiobarbituric acid reactive substances (TBARS) were determined by alkaline hydrolysis of protein bound aldehydes from plasma using an UHPLC system (Agilent Technologies 1290 Infinity) and malondialdehyde (MDA) as standard^56^. The levels of free 4-HNE were determined using a method based on serum samples derivatization in accordance to the procedure suggested^57^, using GC MSMS analysis (Agilent Technologies 7000 A GC/MS Triple Quad direct interfaced with a gas chromatograph GC 7890 A). Plasma oxidized LDL (oxLDL) concentration was determined with Mercodia competitive ELISA kit (Uppsala, Sweden). The measurement of MDA and 4-hydroxy-2-nonenal (4-HNE) associated to apoAI, protein in HDL subpopulations was performed by WB using specific antibodies (see Reagents section). The levels of conjugated dienes in the HDL subfractions were determined by measuring the absorbance at 234 nm.

### Cell culture and *in vitro* tests for HDL function

All experiments to evaluate the HDL function used human umbilical vein endothelial cells (EA.hy926 cell line) (HEC) from ATCC (Manassas, VA, USA), grown after the manufacturer instructions. At confluence, after 6 h starvation (exposure to the serum free medium), HEC were pre-incubated with 80 µg protein/ml HDL_2_ or HDL_3_ without fetal calf serum, for 18 h. Cells were further exposed to additional 10 µM tumor necrosis factor (TNFα). In parallel, cells without HDL pre-incubation were exposed to TNFα in the same condition. After 6 h, the culture media were collected and cells were harvested by lysing them with radio-immunoprecipitation assay (RIPA) buffer. The protein level in cell lysates was quantified by bicinchoninic acid assay following the manufacturer instructions (Sigma-Aldrich Co., MO, USA).

### Western blot analysis

Equal volumes of HDL sub-fractions or culture medium and 40 µg total protein of EC lysate were loaded on a 8/12% SDS-PAGE, transferred to nitrocellulose membrane, blocked with 5% skimmed milk for 1 h, then incubated for 16 h with the primary antibody of the following: ceruloplasmin, 4-HNE-apoAI and MDA-apoAI adducts for HDL subfractions and VCAM-1, ADAM17, MCP-1, and CRP for EC lysate/culture medium samples. Specific antibodies were used as mentioned in Reagents section. The immunostaining detection was made by using the ECL kit (AppliChem GmbH, Darmstadt, Germany) in an LAS4000 analyzer and densitometry of the blots was performed with ImageQuant TL 7.0 software (both from GE Healthcare Bio-Sciences AB, Uppsala, Sweden). The relative level of 4-HNE-apoAI adducts in SA and ACS was expressed as percentage relative to values from N subjects. The protein expression measured in EC was estimated relative to β-actin, and the proteins secreted to the culture medium were expressed relative to cellular protein (BCA assay). The WB images were processed by cropping the gel lanes and using dividing lines where the order of the samples on WB was different that in the final graph.

### Statistical analysis

Statistical software SPSS for Windows v21.0 (IBM SPSS, IBM Ireland, Dublin, Ireland) and GraphPad Prism 5.0 (GraphPad Software Inc., San Diego, CA, USA) were used. The continuous distributed quantitative variables were expressed either as means ± standard deviation (SD, for small datasets <10 values, such as HDL- biochemical composition and cell culture data) or as means ± standard error of the mean (SEM, for large datasets related to patients’ plasma); these data were analysed by two-tailed Oneway ANOVA with Least Significant Difference (LSD) Post-hoc test (for the plasma data in the three groups of subjects) or by Independent Student’s T-test with Levene’s test for equality of variances (for differences between HDL biochemical composition, and for cell culture data for two groups of datasets). Patients’ biochemical data were tested for normal distribution with Shapiro-Wilk test. Crosstabs analysis with chi-squared (χ^2^) function was performed to evaluate the differences between binary logistic data (gender, obesity, diabetes, hypertension, medication). Linear regression curves were plotted for scatterplots between continuous distributed parameters with parametric bivariate correlation analysis performed using the Pearson’s function and its corresponding p-value. A binary logistic regression model (BLR) was performed with the enter iteration method, considering SA group as reference category, and ACS groups as risk category, with serum PON1 activity and mass protein, TBARS, MPO and apoAI levels as covariates, with adjustments for age and gender. The threshold for statistical significance was set to 5% (p-values lower than 0.05).

### Data availability

We state that the materials, data and associated protocols used in the present work will be made promptly available to readers without undue qualifications in material transfer agreements. We disclose no restriction on the availability of materials or information in this manuscript.

## Electronic supplementary material

Supplementary Information
