# Supplementary material for: Dysfunctional high-density lipoproteins have distinct composition, diminished anti-inflammatory potential and discriminate acute coronary syndrome from stable coronary artery disease patients
Source: Sci Rep. 2017 Aug 4;7:7295. doi: 10.1038/s41598-017-07821-5 (PMC5544737; doi:10.1038/s41598-017-07821-5)
Supplement: Supplementary file 1 — Supplementary Information [file 41598_2017_7821_MOESM1_ESM.pdf]

Title

Dysfunctional high-density lipoproteins have distinct composition, diminished anti-inflammatory potential and discriminate acute coronary syndrome from stable coronary artery disease patients

Authors:

Mihaela G. Carnuta[1#], Camelia S. Stancu[1#], Laura Toma[1], Gabriela M. Sanda[1], Loredan S. Niculescu[1], Mariana Deleanu[1,3], Andreea C. Popescu[2], Mihaela R. Popescu[2], Adelina Vlad[4], Doina R. Dimulescu[2], Maya Simionescu[1], Anca V. Sima[1*]

1Institute of Cellular Biology and Pathology “Nicolae Simionescu” of the Romanian Academy, Bucharest Romania

2Cardiology Clinic, Elias University Hospital, Bucharest Romania

3University of Agronomical Sciences and Veterinary Medicine Bucharest, Faculty of Biotechnology, Bucharest, Romania

4Physiology Department, "Carol Davila" University of Medicine and Pharmacy, Bucharest, Romania

# These authors contributed equally to this work

*Corresponding author:

Anca V. Sima, PhD

Institute of Cellular Biology and Pathology “Nicolae Simionescu”

8, B.P.Hasdeu Street, 050568 Bucharest, Romania

Phone: +4021 319 4518 / Fax: +4021 319 4519 / E-mail: anca.sima@icbp.ro

Supplementary Information

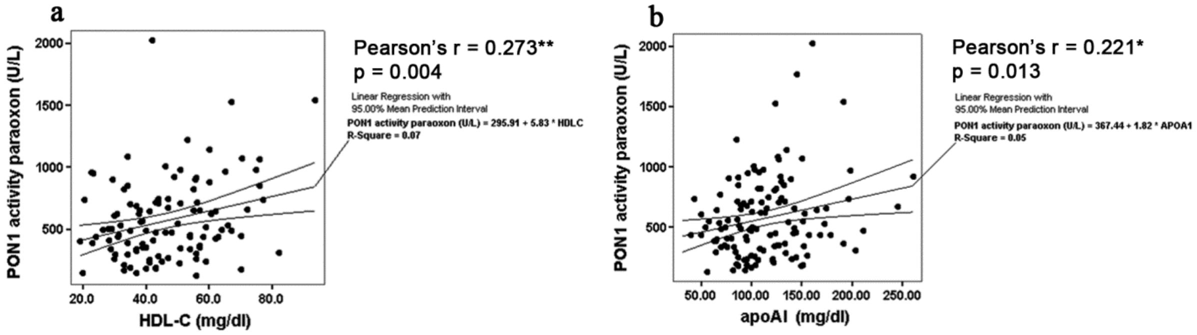

Figure S1. Linear regressions and Pearson's bivariate correlations between paraoxonase 1 (PON1) activity and HDL-cholesterol (HDL-C) (**a**) or apolipoprotein AI (apoAI) (**b**) in sera of CAD patients. *p<0.05, **p<0.01.

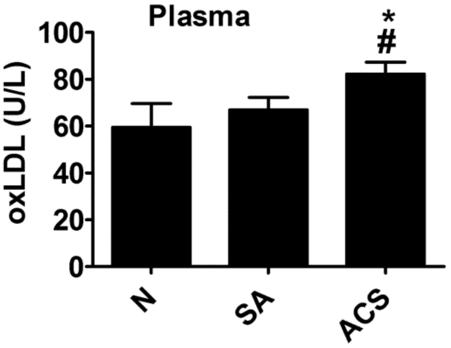

Figure S2. The levels of oxidized LDL (oxLDL) measured in the plasma collected from healthy subjects (N), stable angina patients (SA) and acute coronary syndrome patients (ACS). Data are expressed as means ± SEM and analysed with Independent Student's T-test. *p<0.05 vs. N, #p<0.05, vs. SA.

Table S1. Binary logistic regression for discrimination between ACS and SA patients based on plasma oxidative markers and HDL-associated parameters.

Binary logistic regression[1]	Prediction accuracy (%)	Chi-square step	p-value step	Chi-square model	p-value model
**Model 1[a]**	67.9	-	-	7.453	0.024*
**Model 2[b]**	67.9	3.605	0.165	11.058	0.026*
**Model 3[c]**	67.9	0.864	0.353	11.922	0.036*
**Model 4[d]**	85.7	2.206	0.137	14.128	0.028*
**Model 5[d]**	82.1	2.513	0.285	16.642	0.034*

[1]Dependent variable: risk group (ACS vs SA); Chi-squared test was done for the parameters coefficients (covariates) in the model (B coefficients); * $p<0.05$.
[a] Covariates (predictors) in the model: PON1 activity, PON1 activity/PON1 protein ratio;
[b] Covariates (predictors) added in step: TBARS/PON1 activity, TBARS/PON1 protein;
[c] Covariates (predictors) added in step 3: MPO/PON1 activity ratio;
[d] Covariates (predictors) added in step 4: apoAI;
[e] Covariates (predictors) added in step 4: age, gender.

Table S2. Individual coefficients of covariates entered in the binary logistic regression for discrimination between ACS and SA patients.

Parameter	B	S.E.	Wald	df	Sig.	Exp(B)
**PON1 activity**	-0.015	0.010	2.206	1	0.137	**0.985**
**PON1 activity /PON1 protein**	0.067	0.049	1.874	1	0.171	**1.069**
**TBARS/PON1 activity**	-0.007	3.218	0.000	1	0.998	**0.993**
**TBARS/PON1 protein**	0.063	0.110	0.329	1	0.566	**1.065**
**MPO/PON1 activity**	7.452	7.412	1.011	1	0.315	**1723.334**
**ApoAI**	0.035	0.025	1.868	1	0.172	**1.035**
**Age**	-0.115	0.096	1.448	1	0.229	**0.891**
**Gender**	-0.862	1.309	.434	1	0.510	**0.422**
**Constant**	-6.036	3.848	2.460	1	0.117	**0.002**

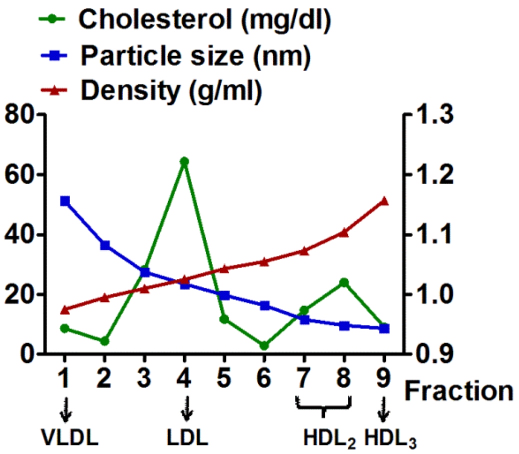

Figure S3. The total cholesterol, particle size and density profiles of the lipoprotein fractions separated by density gradient ultracentrifugation. VLDL – very low-density lipoproteins, LDL – low-density lipoproteins, HDL_2 and HDL_3 – high-density lipoproteins subfractions.

Unprocessed western blot images

Figure 4a

HDL2 - 4-HNE-apoAI

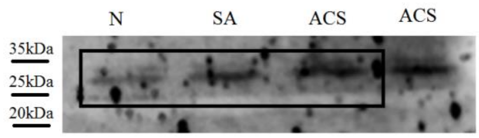

HDL3 - 4-HNE-apoAI

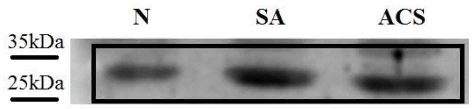

Figure 4b

HDL2 - MDA-apoAI

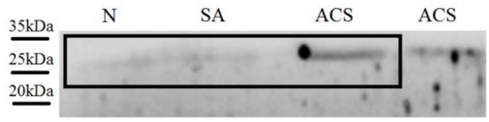

HDL3 - MDA-apoAI

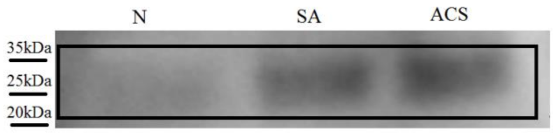

Figure 4c - Ceruloplasmin

HDL2 HDL3

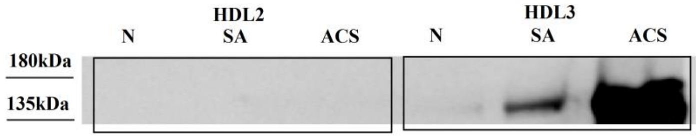

Figure 5a

HDL2 – VCAM-1

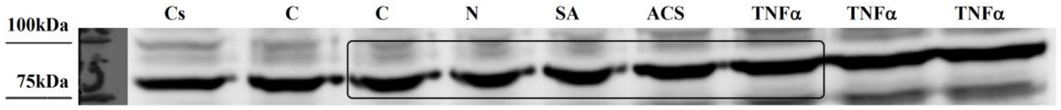

β-actin

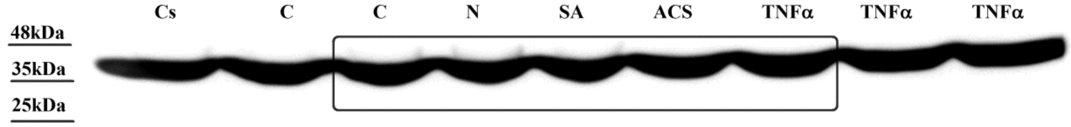

HDL3 - VCAM-1

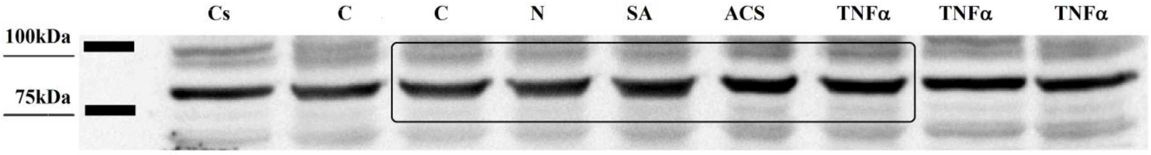

β-actin

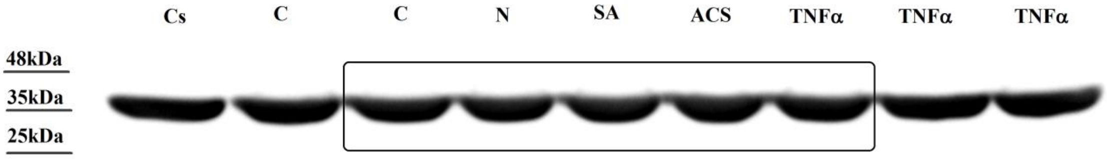

Figure 5b

HDL2 - sVCAM-1

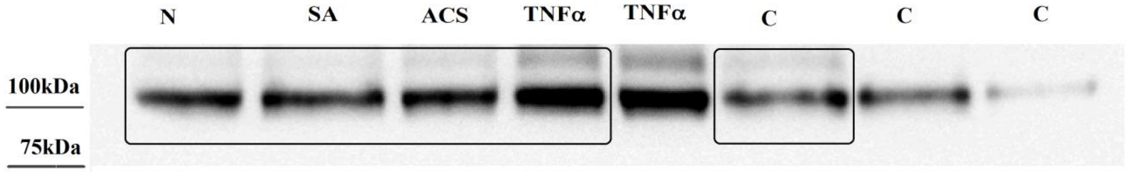

HDL3 - sVCAM-1

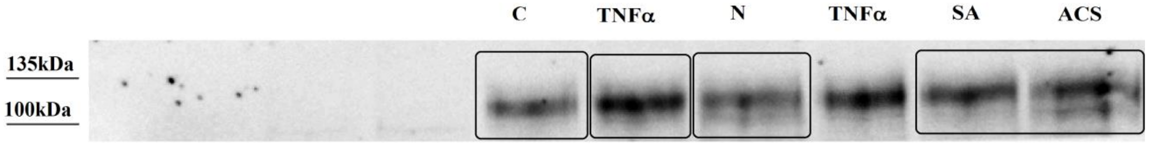

Figure 5c and d

HDL2 – ADAM17

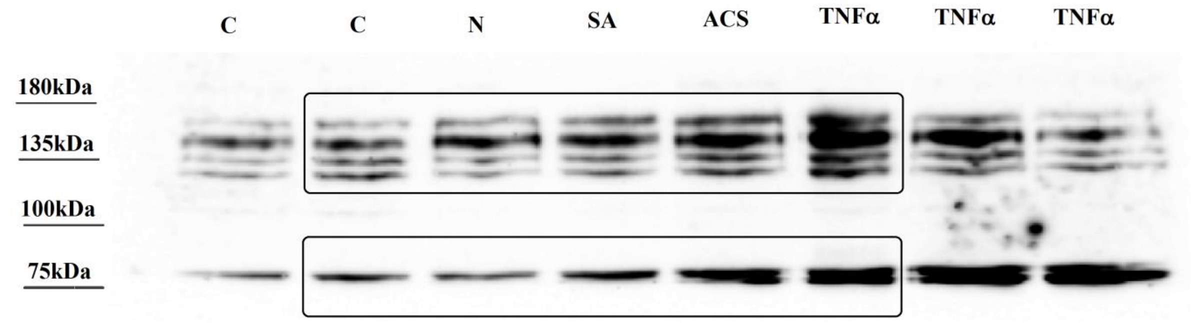

β-actin

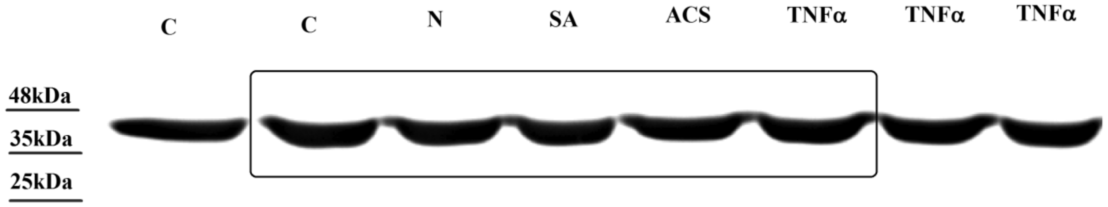

HDL3 – ADAM17

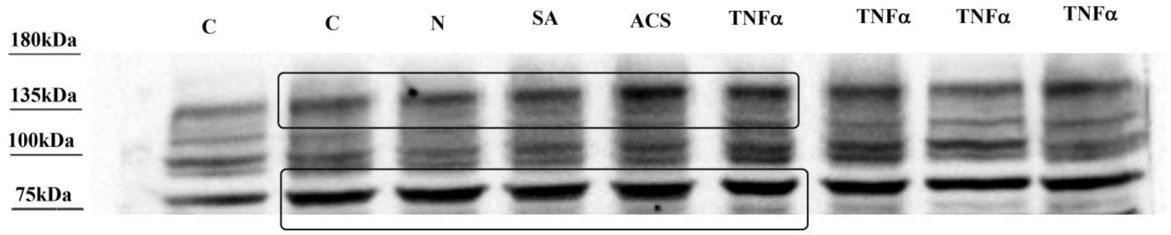

β-actin

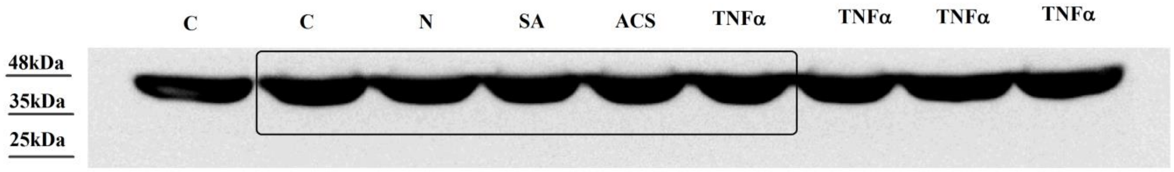

Figure 5e

HDL2 – MCP-1

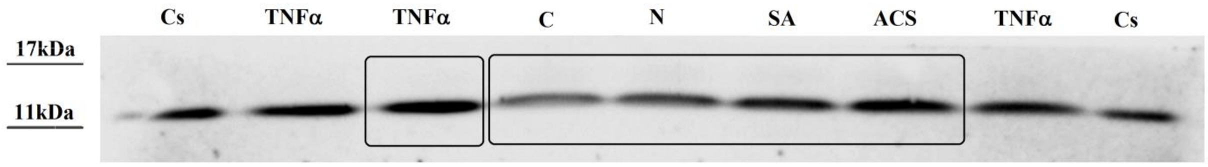

HDL3 – MCP-1

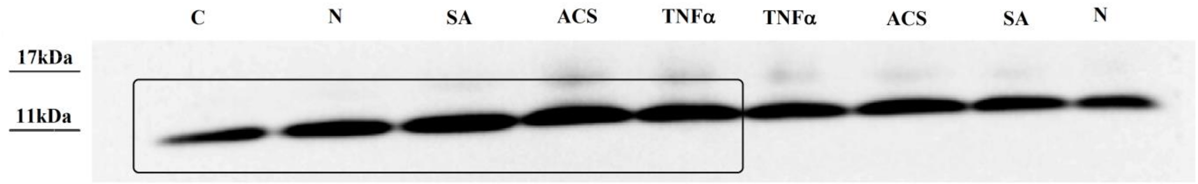

Figure 5f

HDL2 - CRP

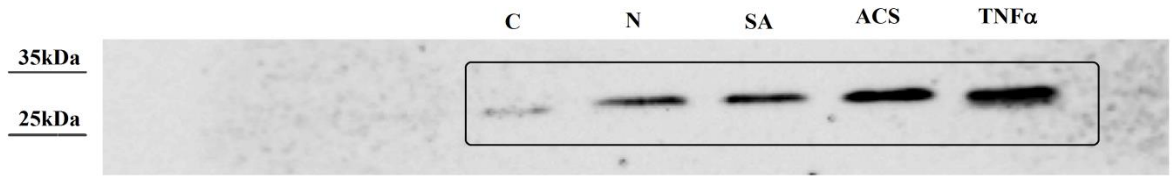

HDL3 - CRP

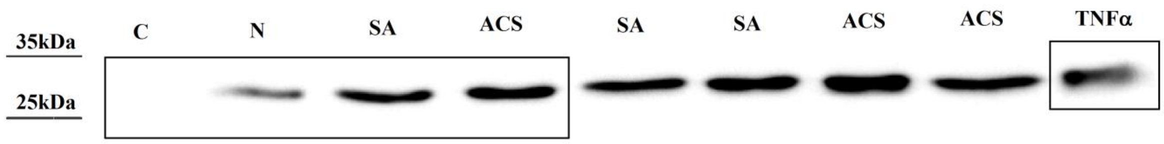

Cs – endothelial cells after 24h starvation and unstimulated with TNFα
